# Psychological benefits of the ASSYST-G protocol for individuals affected by the cold drop meteorological event

**DOI:** 10.3389/fpsyg.2026.1906948

**Published:** 2026-10-21

**Authors:** Chus Taboada Costas, Rosa J. Molero Mañes, María F. Rabito-Alcón, Milagros Molero-Zafra, Francisco Alfonso Burgos-Julián, Cristina Cortés Viniegra, Anabel González Vázquez

**Affiliations:** 1 Centro de Psicología y Trauma ChusTaboada, A Coruña, Spain; 2 EMAFi, Valencia, Spain; 3 Departamento de Psicología y Salud, Universidad a Distancia de Madrid, Madrid, Spain; 4 Universitat de Valencia, Valencia, Spain; 5 Vitaliza, Pamplona, Spain; 6 Instituto Médico Imaya, A Coruña, Spain

**Keywords:** acute stress, ASSYST-G, DANA, early intervention, EMDR, group trauma therapy, natural disaster, Psychological First Aid

## Abstract

**Introduction:**

Climate-related disasters, such as the catastrophic DANA event in Valencia (October 2024), present significant public health challenges, leading to acute stress, anxiety, and functional impairment. Early intervention is critical to prevent the transition from acute distress to chronic post-traumatic stress disorder. This study aimed to evaluate the effectiveness of the Acute Stress Syndrome Stabilization—Group Protocol (ASSYST-G), an EMDR-based intervention rooted in the Adaptive Information Processing model, in reducing subjective distress and traumatic symptoms among survivors.

**Methods:**

A community-based, repeated-measures design was conducted with 570 adults directly exposed to the disaster. Participants were offered up to three 45-min sessions of the ASSYST-G protocol, usually distributed over a 1.5-day period. Symptom severity and Subjective Units of Disturbance (SUD) were assessed at five time points: baseline (T0), after each session (T1, T2, T3), and at a 1-week follow-up (T4) using a validated brief version of the PTSD Checklist for DSM-5 (PCL-5).

**Results:**

Statistical analyses revealed a significant overall decreasing trend in trauma-related symptoms across the ordered assessment occasions (*B* = −0.94, *p* < 0.001). Clinically meaningful improvements were observed in emotional, cognitive, and somatic domains immediately post-intervention, with symptom levels at the 1-week follow-up remaining below baseline levels. The protocol demonstrated high feasibility and tolerability within the high-demand emergency context of the affected municipalities.

**Discussion:**

The findings suggest that ASSYST-G may represent a highly effective, scalable, and time-efficient approach for early trauma intervention. The observed reduction in acute distress is consistent with theoretical models proposing that EMDR-based interventions may contribute to the regulation of sympathetic nervous system hyper activation and facilitate adaptive information processing; however, these mechanisms were not directly examined in the present study and should therefore be considered hypothetical. Overall, the findings are consistent with previous evidence supporting the use of EMDR group protocols and highlight their potential public health value as an early intervention strategy to reduce post-traumatic symptoms following mass trauma events. Future research should examine the long-term stability of these outcomes and investigate the underlying mechanisms of action across different disaster contexts.

## 1 Introduction

The global increase in the frequency and intensity of climate-related disasters represents a growing challenge for public health (IPCC., 2023). Beyond their physical and economic consequences, these events are associated with a significant increase in psychological morbidity. Exposure to natural disasters has been linked to a wide range of psychopathological manifestations, including acute stress disorder, posttraumatic symptoms, anxiety, depression, and dissociation (Morganstein and Ursano, 2020). In the absence of early intervention, these responses may evolve into persistent disorders, such as posttraumatic stress disorder (PTSD), and lead to long-term functional impairment (Bisson et al., 2023).

On October 29, 2024, the Valencian Community (Spain) was affected by a severe episode of *Depresión Aislada en Niveles Altos* (DANA; isolated high-level depression), a meteorological phenomenon characterized by extreme rainfall and flooding. The resulting floods caused extensive damage to critical infrastructure, forced evacuations, and a sustained threat to public safety. In this context, individuals were exposed to core traumatic elements, including intense fear, helplessness, and loss of control (Shapiro, 2017). Such scenarios require early psychological interventions that are scalable, efficient, and evidence-based in order to mitigate the mental health impact at the community level.

Eye Movement Desensitization and Reprocessing (EMDR) therapy has extensive empirical support for the treatment of trauma-related symptoms [WHO, 2013]. Based on the Adaptive Information Processing model, EMDR posits that psychopathology may arise when adverse experiences are inadequately processed and remain dysfunctionally stored in memory networks (Shapiro, 2017). Bilateral stimulation has been associated with the reprocessing of these memories, facilitating their integration and reducing the emotional activation linked to them (Cagaoan et al., 2023; Suara and Retnaningsih, 2023).

In traumatic contexts, several group-based early intervention protocols have been developed to provide rapid and efficient psychological support (Jarero et al., 2011; Jarero and Artigas, 2012, 2022). Among these, the Acute Stress Syndrome Stabilization for Groups (ASSYST-G) protocol stands out as a structured intervention specifically designed for the immediate reduction of symptoms in both acute and ongoing posttraumatic stress contexts (Jarero and Artigas, 2021, 2022). This protocol employs self-administered bilateral stimulation (e.g., the “Butterfly Hug”) within a structured group format, allowing simultaneous intervention with large numbers of participants while maintaining procedural consistency (Girianto et al., 2021).

From a neurobiological perspective, this type of intervention has been proposed to contribute to the regulation of autonomic activation associated with traumatic stress, thereby facilitating reductions in intrusive symptomatology and promoting the restoration of psychophysiological balance (Mainthow et al., 2025). Numerous studies have reported promising outcomes following the implementation of ASSYST-G and related ASSYST protocols (Becker et al., 2021; Carretero et al., 2023; Magalhães et al., 2022, 2023; Mainthow et al., 2022, 2023, 2025; Martínez Cáceres and Premuda-Conti, 2024; Miteva et al., 2025; Ross et al., 2024; Smith et al., 2023; Smyth-Dent et al., 2021). However, despite these encouraging findings, a critical gap remains in the literature regarding the empirical validation of group-based early EMDR interventions deployed during the immediate, acute phase of large-scale, sudden-onset meteorological disasters such as severe flash flooding (Jarero and Artigas, 2022; Miteva et al., 2025). Nevertheless, empirical evidence regarding its effectiveness in the aftermath of large-scale and recent natural disasters remains limited.

The aim of the present study is to evaluate the psychological benefits associated with the implementation of the ASSYST-G protocol in a cohort of individuals affected by the DANA-related floods across a large part of the province of Valencia (Spain). Using a repeated-measures design, the study examines changes in traumatic stress symptoms and subjective distress from baseline assessment to a follow-up conducted between seven and 10 days after the intervention, with the objective of determining the effectiveness and feasibility of this early intervention within the context of a large-scale climate-related emergency.

## 2 Material and methods

### 2.1 Participants

Participant recruitment followed a voluntary self-referral procedure coordinated through the reference city halls and local emergency administration channels in the affected municipalities. Community members experiencing distress symptoms secondary to the flood event registered voluntarily to receive psychological support.

Inclusion criteria were: (a) being aged 18 years or older; (b) having been directly affected by the DANA floods; (c) experiencing self-reported acute psychological distress stemming from the event; and (d) providing informed consent to participate in the intervention and evaluation. Exclusion criteria included: (a) presence of severe, unmanaged psychiatric conditions (e.g., active psychosis) that would impede participation in a group format; (b) severe cognitive impairment; and (c) high immediate suicide risk requiring emergency individual psychiatric referral.

To better characterize the sample according to disaster exposure severity, participants were classified based on their subjective report of impact into three distinct groups: individuals reporting exclusively material damage and losses (subjectively evaluated by each participant); those reporting personal losses, including physical injury, bereavement, or direct life threat to oneself or loved ones (conceptualized as having a higher potential severity of posttraumatic symptomatology due to the acute threat to physical and personal integrity); and finally, a third cohort comprising professional and volunteer first responders, civil protection members, law enforcement officers, and emergency rescue workers who were actively deployed during the immediate aftermath of the disaster.

The final sample consisted of 570 adults exposed to the meteorological event. The mean age was 45.06 years (SD = 17.87). Regarding gender distribution, 75.61% of participants were women and 24.39% were men. Concerning participants' roles, 81.16% belonged to the general population and 18.84% were volunteers. With respect to reported losses, these were primarily material losses (80.70%), followed by other types of losses (11.23%) and personal losses (8.07%). The sample included participants from several affected municipalities, with the largest proportions residing in Sedaví (29.35%), Catarroja (27.20%), and Alfafar (21.72%) (see Table 1).

**Table 1:** Geographic origin and occupational sector distribution. Frequencies and valid percentages of categorical variables.

Variable	Category	Frequency (*n*)	Valid percent-age (%)
Population	Sedaví	150	29.35
	Catarroja	139	27.20
	Alfafar	111	21.72
	Aldaia	58	11.35
	Massanassa	28	5.48
	Benetússer	17	3.33
	Paiporta	8	1.57
Type of group	Citizens	461	81.16
	Volunteers	107	18.84
Losses	Material losses	460	80.70
	Other losses	64	11.23
	Personal losses	46	8.07
Gender	Female	431	75.61
	Male	139	24.39
Occupational sector	Retired/Pensioners	66	11.58
	Public/community sector	62	10.88
	Education and training	57	10.00
	Health and social care	53	9.30
	Retail and customer service	51	8.95
	Unknown/unclassifiable	49	8.60
	Professional occupations	49	8.60
	Homemakers	48	8.42
	Students	47	8.25
	Technical and skilled trades	46	8.07
	Administrative services	42	7.37
	Sedaví	150	29.35
	Catarroja	139	27.20
	Alfafar	111	21.72
	Aldaia	58	11.35
	Massanassa	28	5.48
	Benetússer	17	3.33
	Paiporta	8	1.57
	Other/missing	59	10.35%
	General public	461	81.16
	Volunteer	107	18.84
Losses	Material	460	80.70
	Other	64	11.23
	Personal	46	8.07
Gender	Female	431	75.61
	Male	139	24.39
Professional sector	Retirees/Pensioners	66	11.58
	Public and community sector	62	10.88
	Eduactiona and training	57	10.00
	Health and social care	53	9.30
	Commerce and customer service	51	8.95
	Unknown/unclassifiable	49	8.60
	Liberal psofessions	49	8.60
	Homemakers	48	8.42
	Students	47	8.25
	technical and stalled trades	46	8.07
	Administrative services	42	7.37

Valid percentages were calculated using available cases per variable. For occupational sector, missing data were proportionally imputed to reach a valid N = 570.

### 2.2 Instruments and measures

Posttraumatic symptomatology was assessed using the PTSD Checklist for DSM-5 (PCL-5; Weathers et al., 2013) in its 4-item short form (PCL-5-Short Form; Zuromski et al., 2019), given the specific demands of field-based research and its demonstrated suitability for use in similar populations (Miteva et al., 2025; Price et al., 2016; Zuromski et al., 2019). This brief version is derived from the original 20-item PCL-5 (Weathers et al., 2013) and was developed to provide a concise yet psychometrically robust screening tool for DSM-5 PTSD symptomatology. The four items represent the core PTSD diagnostic clusters, assessing key symptoms such as intrusive thoughts, avoidance, hyperarousal, and negative alterations in mood and cognition. Each item is rated on a Likert scale ranging from 0 (*not at all*) to 4 (*extremely*), yielding a total score ranging from 0 to 16, with higher scores indicating greater symptom severity.

The PCL-5 has demonstrated adequate psychometric properties across diverse populations (Blevins et al., 2015; Bovin et al., 2016). In the present sample, the scale showed adequate internal consistency [α = 0.77, 95% CI (0.74, 0.80)]. The total score was used as a continuous variable to estimate psychological distress across five assessment time points (T0–T4). Given the emergency context and the repeated-assessment design, the administration instructions were adapted only with respect to the temporal reference period. At the initial assessment (T0), participants rated the severity of the assessed symptoms from the time of the traumatic event until the initial assessment. At subsequent assessments (T1–T4), participants rated the severity of the same symptoms with reference to the period since the previous assessment. The content of the four items and the response format remained unchanged across all assessment points. This approach was intended to capture short-term changes in symptom severity during the intervention and immediate follow-up period, while maintaining consistent item content and response options across assessments.

### 2.3 Procedure and study design

The choice of the ASSYST-G protocol for this intervention was driven by specific clinical, logistical, and psychotherapeutic rationales relevant to disaster mental health deployment. First, ASSYST-G is a structured, group-based adaptation of EMDR designed specifically to reduce acute physiological and emotional distress shortly after a traumatic event, thereby preventing the consolidation of posttraumatic pathology (Jarero and Artigas, 2021). Second, its group format provides an efficient and scalable delivery model, enabling clinical teams to reach a high volume of disaster-affected individuals simultaneously under conditions of severe resource constraints (Becker et al., 2021). Third, unlike traditional trauma-focused group therapies, ASSYST-G utilizes self-administered bilateral stimulation (e.g., the Butterfly Hug) without requiring verbal exposure or narrative disclosure of the traumatic memory within the group, thereby minimizing the risk of vicarious traumatization and protecting participant privacy (Jarero and Artigas, 2022). Finally, the protocol's focus on sensory processing and autonomic regulation makes it particularly suited for the early peri-traumatic period, where cognitive processing may be impaired by acute distress (Mainthow et al., 2025).

A longitudinal repeated-measures design was employed to assess changes in symptomatology following the ASSYST-G intervention. Assessments were conducted at five occasions: T0, corresponding to the pre-intervention baseline assessment; T1, T2, and T3, conducted immediately after the first, second, and third intervention sessions, respectively; and T4, a short-term follow-up assessment conducted approximately 1 week after the participant's final session. Under the usual intervention schedule, the first two sessions were conducted on Saturday morning and Saturday afternoon, and the third session on Sunday morning, although some organizational variations occurred across municipalities.

Participants differed in the number of sessions completed and assessments available because of variations in implementation across municipalities and participant availability. Of the 570 participants included in the analysis, 569 provided data at T0, 544 at T1, 355 at T2, and 199 at T3. Because attendance was not recorded independently of the corresponding post-session assessment, these figures refer to the number of participants assessed at each occasion rather than to separate attendance counts. Accordingly, T1–T3 represent consecutive session-linked assessment occasions, although not all participants completed or were assessed following all three sessions.

Given the unbalanced nature of the design, with a varying number of observations per participant, a linear mixed-effects modeling approach was selected for the analysis of the longitudinal data (see Statistical Analysis section). Analyses were conducted according to the intention-to-treat principle in order to accommodate missing data and minimize potential bias associated with participant attrition and incomplete follow-up assessments.

### 2.4 Statistical analysis

Statistical analyses were conducted using R software version 4.5.1 (R Core Team, 2025). To characterize the sample, absolute frequencies (*n*) and percentages (%) were calculated for categorical variables. Missing data, which were distributed across the different assessment time points (see Table 1), were addressed through multiple imputation procedures prior to conducting the longitudinal analyses.

Longitudinal data were analyzed using linear mixed-effects models (LMMs) fitted with the *lme4* package (Bates et al., 2015). This approach is well suited for repeated-measures data with an unequal number of observations per participant. Time was coded as an ordered assessment-occasion variable representing the sequence of session-linked assessments rather than exact elapsed time measured in hours or days. Accordingly, the time coefficient represents the average change associated with progression from one ordered assessment occasion to the next, rather than change per fixed unit of chronological time. The model included time as a fixed-effect predictor and random intercepts and slopes for each participant, allowing the estimation of inter individual variability in baseline symptom levels and patterns of change across the ordered assessment occasions. The model was specified as follows:


$$\begin{array}{l} \text{PCL}\text{-}5_{ij}\sim {\text{Time}}_{j}+\left(1+{\text{Time}}_{j}|{\text{id}}_{i}\right) \end{array}$$ (1)

*p*-values were calculated using the Satterthwaite approximation implemented in the *lmerTest* package (Kuznetsova et al., 2017), and model coefficients were standardized using the *parameters* package (Lüdecke et al., 2020). Effect sizes were reported as standardized beta coefficients (β) and interpreted according to Cohen (1988) guidelines: 0.10 (small), 0.30 (moderate), and 0.50 (large).

### 2.5 Ethics statement

All study procedures were reviewed and approved by the Research and Teaching Ethics Committee of the Faculty of Psychology and Health Sciences at the Universidad a Distancia de Madrid (UDIMA) (approval code: PSIP20201220-3). The study was conducted in accordance with the ethical principles outlined in the Declaration of Helsinki. Confidentiality was ensured throughout the research process, and informed consent was obtained from all participants prior to their participation in the study.

## 3 Results

### 3.1 Sample characteristics and descriptive statistics

The final analyzed sample consisted of 570 participants. The mean age was 45.06 years (SD = 17.87). Regarding gender distribution, 75.61% (*n* = 431) of the sample were women and 24.39% (*n* = 139) were men.

With respect to exposure to the DANA meteorological event, most participants reported material losses (80.70%), followed by other types of losses (11.23%) and personal losses (8.07%). The sample primarily consisted of members of the general population (81.16%), with a smaller proportion of volunteers (18.84%). The distribution of participants by geographical location and professional sector is presented in Table 1.

### 3.2 Reliability and missing data analysis

The PCL-5 demonstrated adequate internal consistency in this sample [α = 0.77, 95% CI (0.74, 0.80)]. The proportion of missing data was 0.17% at baseline (T0), increasing to 65.09% at T3 and reaching 42.28% at follow-up (T4), as shown in Table 2. To minimize potential bias associated with missing information, missing data were handled using multiple imputation procedures prior to conducting the longitudinal analyses.

**Table 2:** Frequency and proportion of missing cases at each time point.

Time point	Missing cases (*n*)	Proportion (%)
T0	1	0.18
T1	26	4.56
T2	215	37.72
T3	371	65.09
T4	241	42.28

### 3.3 Intervention effectiveness: linear mixed-effects model (full sample)

The fixed-effects analysis revealed a significant effect of time on PCL-5 scores, indicating an average decreasing trend in posttraumatic symptomatology across the ordered assessment occasions (B = −0.94, SE = 0.04, *t* (568.93) = −25.81, *p* < 0.001). The standardized coefficient was β = −0.44 [95% CI (−0.47, −0.40)], indicating a moderate-to-large effect size according to Cohen (1988) criteria. The results of the linear mixed-effects model for PCL-5 scores in the full sample are presented in Table 3.

**Table 3:** Linear mixed-effects model results for PCL-5 scores (full sample).

Parameter	*B*	SE	df	*t*	*P*
**Fixed effects**					
(Intercept)	6.08	0.16	568.99	38.68	< 0.001
Time	−0.94	0.04	568.93	−25.81	< 0.001
**Random effects**	Variance	SD			
ID (Intercept)	9.97	3.16			
ID (Time)	0.08	0.28			
Residual	6.86	2.62			

Mean PCL-5 scores decreased from 6.95 (SD = 4.09) at T0 to 2.38 (SD = 3.45) at T3. At follow-up (T4), the mean was 3.52 (SD = 3.67), remaining below baseline levels. SE, standard error; SD, standard deviation; df, degrees of freedom.

## 4 Discussion

### 4.1 Main findings and intervention effectiveness

The present study aimed to evaluate the psychological effects of the early implementation of the ASSYST-G protocol following the DANA floods in the province of Valencia. The results showed a significant overall decreasing trend in posttraumatic symptomatology across the ordered assessment occasions. Specifically, the mean PCL-5 score decreased from 6.95 at baseline to 3.52 at follow-up, with a moderate-to-large effect size (β = −0.44). These findings suggest that the intervention was associated with a clinically meaningful improvement over a relatively short period, consistent with previous research (Becker et al., 2021; Carretero et al., 2023; Magalhães et al., 2022, 2023; Mainthow et al., 2022, 2023, 2025; Martínez Cáceres and Premuda-Conti, 2024; Miteva et al., 2025; Ross et al., 2024; Smith et al., 2023; Smyth-Dent et al., 2021). However, given the study design, the findings should be interpreted cautiously with respect to causal inference.

Furthermore, initial symptom severity and treatment response trajectories were influenced by disaster exposure parameters. Participants reporting personal losses (e.g., bereavement, personal injury, or direct life threat) exhibited higher baseline posttraumatic distress compared to those who experienced exclusively material damage or served as first responders/volunteers. Nonetheless, the ASSYST-G protocol demonstrated consistent treatment gains across all exposure categories and participant subgroups (i.e., material loss, personal loss, emergency/volunteer personnel), and geographical locations, highlighting its broad applicability in heterogeneous disaster-affected populations.

From the Adaptive Information Processing (AIP) framework, bilateral stimulation has been proposed to facilitate the reprocessing of traumatic experiences and reduce associated emotional activation (Shapiro, 2017). In line with this framework, the ASSYST-G protocol was specifically designed to target core components of the traumatic experience, particularly intrusion-related symptoms, within a structured group format (Jarero and Artigas, 2022).

### 4.2 Previous research context and novelty

The literature on EMDR Early Interventions (EEIs) has expanded substantially in recent years. Systematic reviews suggest that the application of brief EMDR protocols during the first months following trauma may significantly reduce the risk of symptom chronicity (Ballesteros et al., 2025). However, the novelty of the present study lies in its large-scale and near-immediate implementation during an ongoing crisis.

Unlike longer and more intensive interventions, ASSYST-G occupies an intermediate position between Psychological First Aid (PFA) and more comprehensive psychotherapeutic approaches, within a stepped-care framework guided by symptom trajectories. Whereas PFA primarily focuses on safety, stabilization, and the provision of basic needs, ASSYST-G directly targets posttraumatic symptomatology, particularly intrusion-related symptoms, through a structured and efficient group-based format (Jarero et al., 2006, 2011, 2017; Wang et al., 2024).

This study contributes one of the largest documented samples in Spain examining the use of EMDR group protocols following a climate-related disaster, providing relevant evidence regarding their applicability in contexts characterized by high service demand and limited resources.

### 4.3 integration within current knowledge and advancement of perspectives

The present findings contribute to the disaster mental health literature by demonstrating that evidence-based psychological interventions can be implemented on a large scale under real-world emergency conditions. In this regard, the results suggest that the ASSYST-G protocol may facilitate the stabilization of intrusion-related symptoms during the early stages following traumatic exposure.

From a theoretical perspective, EMDR-based interventions have been proposed to contribute to the regulation of trauma-related stress activation and to facilitate adaptive information processing (Jarero and Artigas, 2012; Jarero et al., 2006, 2011; Mainthow et al., 2022; Smith et al., 2023). Nevertheless, the mechanisms underlying these effects require further empirical investigation.

A specific adaptation implemented in the present study involved the combined use of self-administered bilateral stimulation (e.g., the Butterfly Hug) together with rhythmic foot movements. This modification was derived from the individual ASSYST format and did not result in any observable difficulties in participant regulation. This approach may be interpreted within working memory taxation models, which propose that dual-attention stimulation contributes to reducing the vividness and emotional intensity of traumatic memories. However, this hypothesis should be examined in future studies. To date, research involving combined stimulation modalities suggests that such approaches are technically feasible and show preliminary signals of effectiveness in small samples, although the current evidence remains insufficient to conclude that multimodal stimulation is clinically superior to a single modality within EMDR interventions (Grifoni et al., 2023; Herkt et al., 2014; Kamińska et al., 2020).

Furthermore, these findings should be considered within the broader context of increasing concern regarding the mental health consequences of climate change. Current evidence indicates that extreme weather events are associated with an elevated risk of posttraumatic symptomatology and related mental health difficulties (Lawrence et al., 2024; Macaluso et al., 2023; Patwary et al., 2024). Within this context, interventions such as ASSYST-G may represent a useful component of public health strategies aimed at mitigating the psychological consequences of climate-related disasters.

### 4.4 Limitations and methodological challenges

Despite the large sample size and the consistency of the findings, several limitations should be considered when interpreting the results.

#### 4.4.1 Participant attrition and missing data

A substantial increase in missing data was observed at later assessment points (e.g., T3). This phenomenon is common in disaster research, where participants' priorities often change over time. To address this limitation, linear mixed-effects models were employed, as these approaches can accommodate unbalanced data structures and reduce bias associated with participant attrition. Nevertheless, the potential influence of missing data on the findings cannot be completely ruled out.

#### 4.4.2 Lack of a control group

The study design did not include a control group, limiting the ability to establish causal relationships between the intervention and the observed changes. Although previous studies suggest that EMDR early interventions may outperform natural recovery processes (Shapiro and Maxfield, 2019), the findings of the present study should be interpreted cautiously in this regard.

#### 4.4.3 Adaptation of the symptom assessment procedure

Although the content of the four selected PCL-5 items remained unchanged, the administration instructions were adapted by modifying the temporal reference across repeated assessments in order to monitor symptom evolution during an intensive early intervention delivered in a humanitarian emergency context. Consequently, these repeated assessments should be interpreted as measures of short-term symptom monitoring rather than as repeated diagnostic applications of the original PCL-5. Although this approach reduced participant burden and increased the feasibility of conducting repeated assessments in a large-scale disaster response, it differs from the standard administration procedure for which the instrument was originally validated and should therefore be considered when interpreting the findings.

#### 4.4.4 Limited follow-up period

The follow-up period was relatively short (7–10 days), preventing conclusions regarding the stability of the observed effects over the medium and long term. Future studies should incorporate longer longitudinal follow-up assessments to determine the persistence of treatment-related benefits. In addition, future research should examine whether these findings can be replicated using outcome measures specifically validated for repeated assessments over very short time intervals following traumatic events.

### 4.5 Future directions and theoretical implications

The findings of the present study suggest that the ASSYST-G protocol may contribute to the early reduction of posttraumatic symptomatology, raising important hypotheses regarding its potential mechanisms of action. Future research should therefore incorporate additional outcome measures, including stress-related biomarkers, to more precisely examine the processes underlying therapeutic change.

Moreover, the scalability demonstrated in this study highlights the potential for integrating interventions of this nature into broader emergency response strategies, including public health initiatives and civil protection programs. In a context characterized by the increasing frequency of climate-related disasters, the development and implementation of early, efficient, and accessible psychological interventions may play an important role in promoting community resilience and reducing the mental health burden associated with large-scale traumatic events.

## Data Availability

The datasets generated and/or analyzed during the current study are not publicly available due to patient confidentiality and ethical restrictions. However, anonymized data are available from the corresponding author upon reasonable request.
